# Yaws in the Western Pacific Region: A Review of the Literature

**DOI:** 10.1155/2011/642832

**Published:** 2011-12-22

**Authors:** Corinne Capuano, Masayo Ozaki

**Affiliations:** ^1^World Health Organization, Office of the WHO Representative for Brunei Darussalam, Malaysia and Singapore, P.O. Box 12550, 50782 Kuala Lumpur, Malaysia; ^2^Department of Epidemiology, Johns Hopkins Bloomberg School of Public Health, 615 N. Wolfe Street, Baltimore, MD 21205, USA

## Abstract

Until the middle of the 20th century, yaws was highly endemic and considered a serious public health problem in the Western Pacific Region (WPR), leading to intensive control efforts in the 1950s–1960s. Since then, little attention has been paid to its reemergence. Its current burden is unknown. This paper presents the results of an extensive literature review, focusing on yaws in the South Pacific. Available records suggest that the region remains largely free of yaws except for Papua New Guinea, Solomon Islands, and Vanuatu. Many clinical cases reported recently were described as “attenuated”; advanced stages are rare. A single intramuscular injection of benzathine penicillin is still effective in curing yaws. In the Pacific, yaws may be amenable to elimination if adequate resources are provided and political commitment revived. A mapping of yaws prevalence in PNG, Solomon, and Vanuatu is needed before comprehensive country-tailored strategies towards yaws elimination can be developed.

## 1. Introduction

Yaws is a nonvenereal infectious disease caused by the bacterium *Treponema pallidum* subspecies *pertenue*. It is mainly transmitted from person to person through direct contact with exudates from early skin lesions of infected people [1]. Yaws is considered a disease of poverty occurring in tropical regions throughout the world with heavy rainfall and high humidity [2]. It is more common in rural and isolated populations where access to health care is often limited [3]. Crowded environments and poor hygiene are also considered as factors facilitating transmission [4, 5]. The disease affects predominantly children younger than 15 years (the peak incidence of clinical manifestations is 2 to 10 years), who serve as the primary reservoir of the disease. The current knowledge is that transmission is by direct contact with infected lesions [2] and that flies, including nonbiting haematophagous ones, can infect skin breaches through their dejecta or regurgitation [6, 7]. Perine et al. [2] reported that a yaws-like treponema was identified in African monkeys and baboons, and more recently Robed et al. [8] mentioned that the genetic analysis of a strain collected from a Guinean baboon demonstrated a close relation to the human strains of yaws. Furthermore, yaws-like infections have been identified in nonhuman primates in Africa, in particular in the Republic of Congo where 17% of a wild gorilla population have been found with typical yaw lesions [7] leading the authors to speculate that yaws infections in gorillas and humans living in tropical rain forests might be due to the same bacterium *Treponema pertenue. *Considering that in humans and gorillas *T. pertenue* spreads by direct contact with infected lesions [7] and that flies also play a role in transmission of the bacterium, a risk of contamination between humans and other primates might exist. These findings argue in favor of a potential role of yaws-infected nonhuman primates in humans' infections. However, there is no evidence of such transmission in the literature, and the actual significance of these findings to human is not known [2, 3, 9]. This debate is interesting and certainly deserves more research to establish if pathogens cross-transmit between humans and primates populations. Nevertheless, for the Western Pacific Region, we did not find evidence of recent resurgence of yaws in countries where frequent encounters between humans and monkeys occur (Cambodia, Malaysia, or Vietnam, e.g.), but we found evidence of recent yaws reappearance in Pacific countries where there is no population of nonhuman primates. Furthermore, India which hosts a large population of monkeys with frequent encounters with humans managed to eliminate yaws. Therefore, the provocative question of whether humans are in fact the reservoir of the disease to other primates might be raised. In the absence of nonhuman primates in the Pacific, humans are the only known reservoir of the disease in this part of the region.

It is estimated that there were 50 to 150 million cases of active yaws worldwide in the early 1950s [3]. A global campaign to control endemic treponematoses, including yaws, was launched in 1952 targeting 46 countries [10]. By the end of 1964, the global campaign, supported by the World Health Organization and the United Nations Children's Fund, successfully reduced the prevalence of yaws by 95% to an estimated 2.5 million cases [4, 10–12]. Yaws surveillance and control activities subsequently became integrated into the primary health care systems of individual countries, where remaining cases were to be identified and treated [10]. Yaws transmission persisted, however, although at low levels, and the passive approach for yaws control under primary health care systems was not efficient in detecting and treating cases remaining in remote and isolated areas of developing countries. In the 1970s, resurgence was reported in many of the formally endemic areas [10]. Despite efforts to renew the commitment to yaws control and to reengage the international community (e.g., World Health Assembly Resolution 31.58 of 1978 on yaws; global and regional meetings in the early 1980s), yaws persisted in many parts of the world, with the largest number of cases found in west and central Africa [13, 14].

In the South Pacific, records show that yaws was highly prevalent prior to mass treatment campaigns carried out in the late 1950s and early 1960s [15]. Following mass treatment campaigns, the number of reported cases dramatically declined, and yaws was considered eliminated in most areas of the South Pacific [16, 17]. Since the late 1970s, however, suspected cases of yaws were reported in various areas of Papua New Guinea (PNG) [18–23], the Solomon Islands [16, 24–26], and Vanuatu [17, 27, 28]. While available records suggest that these countries remain endemic to this date, the extend of the current burden due to yaws is not well known [9].

In our twelve-year experience as a field public health expert in the Pacific, we had to deal with yaws in many instances. Over the years, we spent time doing literature researches to address, when they aroused, specific issues linked to yaws control activities in the Pacific, and we often felt that a single document providing all available information together with relevant references would be useful to clinicians and to health workers. In the past three decades, yaws control activities have been localized in a number of areas in the Pacific, and there is an obvious disconnect between regular field observations of yaws coherent with verbal reports from front line health workers mentioning that yaws has never stopped or has reappeared in the past few decades and the patchy and scarce information found in the literature. There is no document providing an overall picture of the current yaws situation in the region, and the literature consulted over the years gave the impression that most activities have been conducted in silos, answering to a sudden and localized resurgence or interest on yaws. Discussions with colleagues and health workers confirmed this overall sense of a heterogeneous approach in the region and the lack of a clear idea of the current situation in regards to a disease that has supposedly been eradicated more than half a century ago but that is regularly encountered in certain parts of the region. It then became clear that a review of all the available information and references for the region would be a useful tool not only to clinicians but also to public health decision makers. A thorough assessment of the yaws situation in the Western Pacific Region (WPR) over the past 60 years and the potential challenges yaws eradication poses today could serve as a reminder for the public health community in the region about this neglected disease. It could stimulate a discussion among regional public health decision makers and pave the way for a coordinated approach in the areas still affected. With this in mind, we reviewed the literature currently available, and we provide in this paper a summary of epidemiological records on yaws in the WPR with a special focus on the Pacific area since the 1980s. It also reviews past control efforts in the region with an emphasis on PNG, the Solomon Islands, and Vanuatu.

## 2. Materials and Methods

In addition to data provided by WHO Member States, we searched on MEDLINE and WHOLIS, the World Health Organization's library database, for relevant articles using the term “yaws.” To identify references specific to the region (primarily South Pacific), the following terms were used: “South Pacific,” “Pacific,” and “Western Pacific” for countries outside the Pacific, and individual country names. Articles published through 27th December 2010 were included. A similar search using the same words was conducted on WHOLIS.

We first reviewed titles and abstracts and then selected articles to be fully reviewed. Some articles from the 1950s to the 1970s in particular were excluded due to the contents being too similar to the ones already referenced or because they were quoted in more recent articles. The whole process identified 53 relevant documents, all of which are referenced in this paper.

An additional search with “endemic treponematosis” and with “endemic treponematoses” was conducted which found 300 references. The search was then limited to “Western Pacific,” “Pacific,” “Asia,” and individual country names in the region together with the term “endemic treponematosis” or “endemic treponematoses.” Only two additional relevant articles were found but were excluded for similar reasons to those explained above.

## 3. Results and Discussion

### 3.1. Past and Current Yaws Control Strategies in the South Pacific Region

#### 3.1.1. Past Efforts

Yaws control during the 1950s and 1960s put a strong emphasis on assessing the population-level prevalence of active and infectious yaws cases. Initial surveys often covered a large proportion of endemic populations and were conducted together with mass or selective treatment using procaine penicillin G with 2% aluminum monostearate (PAM) [15]. For example, in the Cook Islands, 99% of the population were screened in 1960, and in Vanuatu, an initial mass treatment survey conducted in 1958 covered 94% of the indigenous population [15]. The results of these surveys were also utilized to determine appropriate control methods. In most cases, further surveys were conducted to identify and treat previously untreated cases and their contacts [15]. The national control programs were established with the assistance of WHO and UNICEF in most areas of the South Pacific and received technical assistance from an Interregional Treponematosis Advisory Team consisting of a medical officer, a serologist, and a nurse/administrative officer based in the region, in particular for training of national staff members in conducting surveys and diagnostic techniques [29]. Surveys conducted in the 1960s and 1970s showed successful reduction of yaws below detectable levels in most areas of the South Pacific. For example, Geizer reported [15] that only 6 cases were found in Niue in 1957, while the disease was widely spread earlier. According to Geizer, only 3 cases were diagnosed between 1964 and 1984 in Fiji, the last one being in 1983, while initial surveys conducted in 1954 found 10% to 70% prevalence of reactive serology. In Tonga, the last 7 cases were reported in 1976 while 7,452 cases were reported in 1962 when the eradication campaign was launched. Several authors reported that yaws control activities were then gradually integrated with other communicable disease programs into basic health services without providing further details [3, 9, 30]. In 1985, Meheus [31] and Hopkins [32] discussed integration of yaws control activities into Primary Health Care (PHC) interventions. Meheus explains that control of yaws was then relying on several strategies combining population screening with mass treatment, and case finding/diagnosis at consultation with contact tracing/treatment. In addition, professional training and health education were conducted. We did not find concrete examples on how these yaws control activities were introduced, implemented, and integrated in the South Pacific.

Lack of funding associated with weak political commitment and fragile primary health care systems resulted in the continued occurrence of yaws and its poor reporting [9]. By the 1970s and 1980s, sporadic outbreaks were reported in PNG, the Solomon Islands, and Vanuatu, and in most cases, the immediate responses to these outbreaks were recorded and published. However, as Geizer discussed [15] in 1986, information on yaws became scarce once the outbreaks were contained, and very limited information about surveillance activities is currently available for these countries.

#### 3.1.2. Recent Approaches to Yaws Control

In 1984, the WHO recommendations were published [2] in response to resurgence in the 1970s. According to the recommendations, a treatment strategy for yaws control should be based on the prevalence of clinically active yaws. Thus, the prevalence of active case needs to be assessed in a representative sample of a population of interest (e.g., district, province, or country) in order to determine an appropriate treatment policy for the population. The recommendations also suggest that treatment of the entire population (total mass treatment), treatment of all active cases and obvious contacts (contacts being defined as those who have frequent, direct, person-to-person contact with a patient with active yaws lesions) as well as all children under 15 years of age (juvenile mass treatment), or treatment of all active cases and all household and other obvious contacts (selective mass treatment) should be adopted based on the level of prevalence of clinically active yaws in the community (Table 1). In addition, it was indicated that in remote areas where access to health services is limited, total mass treatment may be a more appropriate option even when the population prevalence is less than 10%.

Benzathine benzylpenicillin was recommended in preference to the other forms of penicillin, penicillin aluminium monostearate (PAM) in particular as the serum concentration of penicillin produced by benzathine benzylpenicillin persists above the treponemicidal level much longer than that produced by PAM [2]. The following dosages were recommended:

- 600.000 units for children below 6 years;
- 1.2 million units for children between 6 and 14 years;
- 2.4 million units for children above 14 years and adults.

These recommendations are still applicable today for the control of yaws, and the same treatment using a single dose of benzathine penicillin is still considered the standard treatment for yaws [33]. Tetracycline, erythromycin, or doxycycline can be used for patients with penicillin hypersensitivity [10].

To be successful mass treatments must reach a very high level of coverage. While no studies we reviewed specified the minimum level of coverage to be targeted, Hudson et al. [34] argued that the successful eradication campaigns in the 1950s and 1960s can be in part attributed to the fact that the treatment of the entire treponemal reservoir, including active and latent cases, was achieved. Perine et al. [2] stated that “all contacts of infectious cases must be treated if yaws is to be eliminated from the community” and estimated that 1 to 3% of yaws-affected patients may not be cured by the recommended treatment due to quality issues with the penicillin used or the use of altered penicillin associated with improper storage, reconstitution, or expiration of drugs.

While mass treatment is challenging in terms of logistics and requires high political commitment [17], the low cost of benzathin penicillin and its efficacy make it a valuable option for treating yaws in a population. De Noray et al. [17] estimated the cost of the 2001 mass treatment conducted in SANMA province, Vanuatu, to be USD 1.30 per person. Treatment with penicillin always carries the risk of severe side effects such as anaphylactic shock, and this must be considered when planning such interventions [2, 17].

Based on the WHO recommendations, serological testing is necessary to identify latent or incubating cases. However, in some cases, serological testing during a prevalence survey may not be necessary, since most latent or incubating cases are found in clusters near an infectious case [2]. These would receive treatment as household or obvious contacts (even if they were not identified as cases). Perine et al. [2] indicated that the prevalence of active yaws observed during a survey could be used to estimate the prevalence of seroreactor defined as seroactive yaws cases. Whether or not these estimations apply to the current epidemiology of yaws is not well understood (Table 2).

Most yaws control activities implemented in the South Pacific since the 1990s followed the above-recommended strategies and treatment regimen. However, in 2010, Fegan et al. [28] raised a question on the use of benzathin penicillin, mainly because of its logistical constraints. The same authors suggested exploring the possibility of using an oral treatment such as azithromycin, a macrolide antibiotic with a known efficacy in *T. pallidum* infections and with a long half-life in tissue. Compared to the challenges posed by treatments with benzathine penicillin (logistics, possible allergy, and pain associated with the injection), azythromycin presents some true advantages. Its oral presentation and its efficacy in single-dose administration for the treatment of syphilis in adults [35] make it potentially a more accessible and more acceptable treatment at peripheral level. However, some reservations must be considered. First, macrolide-resistant *T. pallidum* initially identified in groups with high-risk sexual behavior in the United States and Ireland [36] has now been isolated in other countries such as China where Martin et al. [37] report an apparent high prevalence of macrolide resistant *T. pallidum* in Shanghai, the Czech Republic where the prevalence of the mutations responsible for macrolide-resistance is reported to be as high as 20% in clinical specimens [38] or in Canada where 29% of syphilis cases were found to be caused by a strain resistant to azythromycin [39]. The recent identification of macrolide-resistant *T. pallidum *subspecies* pallidum* in several countries raises concerns on the possible independent emergence of multiple strains and on the role selective pressure and other conditions may have played. Furthermore, it raises questions on the impact of these findings on other subspecies of *Treponema pallidum* including *pertenue. *It also allows speculations that similar mutations leading to macrolide resistance might quickly appear with yaws bacterium. These reservations and the consistent success of penicillin in the treatment of syphilis and yaws for more than fifty years strongly argue in favor of benzathine penicillin as the treatment of choice for yaws. 

In 1998, following a study in 39 children with clinical yaws in Karkar Island, PNG, Backhouse et al. [22] reported that while initial clinical and serologic responses to benzathine penicillin were satisfactory in more than 90% of the children, 11 (28%) later showed clinical and/or serologic evidence of relapse or reinfection. These authors concluded in favor of treatment failures due to reduced susceptibility to penicillin because reinfection was unlikely in this particular community and under the circumstances of the study. To our knowledge, this possible reduction in susceptibility or tolerance to benzathine penicillin of *T. pallidum* subsp. *pertenue* has not been further documented, and additional data to support the finding could not be found in the South Pacific.

### 3.2. Diagnosis of Yaws in the South Pacific

In most cases, diagnosis was based on clinical criteria during the 1950s and 1960s [15]. With the disappearance of yaws in a number of countries and the progressive retirement of those who were involved in the eradication campaigns in the 1950s and 1960s, some authors [34] suggested that most health workers have never seen the disease and may not be able to make a clinical diagnosis. However, our review shows that yaws has been most often detected clinically by primary health care workers, for example, in Vanuatu in 2000 [17] and PNG in 1995 [23], who then reported to their local authorities, leading to further investigations and control activities. It is also possible that some of those workers had been involved in the past or more recent control efforts and were capable of recognizing the symptoms. On another hand, a number of skin conditions common in the tropics may appear similar to yaws lesions, and thus, it is possible that some proportion of yaws reported in primary health care settings may in fact be false positive cases. In some instances, clinically identified cases were confirmed serologically. Nevertheless, clinical diagnosis made in recent years may be less specific as the number of primary health care workers experienced in yaws diagnosis is declining, and a large number of active cases identified in recent years are reported as presenting milder symptoms compared to the 1950s [18, 28, 40]. One can speculate about the accuracy of this contention, especially as there is nearly no survey available comparing results of yaws clinical studies in the same area in the 1950s and nowadays. However, for the Western Pacific Region, we found one survey [16] where the authors compare clinical findings from two surveys conducted in 1953 and in 1987 in the same island of the Solomon Islands and found 10% of cases with secondary or tertiary yaws in 1987 compared to 30% in 1953. The reasons for these attenuated clinical manifestations are unclear, but they are likely the consequence of a mix of different factors such as more accessible health systems possibly resulting in earlier diagnosis of infections and a wide use of antibiotics (especially penicillin since the 1950s) which might delay the occurrence of clinical lesions.

Common serological tests currently used in the South Pacific are the Venereal Disease Research Laboratory (VDRL) test and the Rapid Plasma Reagin (RPR) test. Both are sensitive, but they are nontreponemal antigen tests and do not differentiate yaws from other treponemal infections such as syphilis [2, 4]. However, it is important to acknowledge that treponemal tests such as the fluorescent treponemal antibody-absorption enzyme immunoassay (FTA-ABS), the *Treponema pallidum* particle agglutination (TPPA) test, or the enzyme immunoassay (EIA) cannot make such a distinction either. This reality can be of importance as it represents a particular challenge in countries where both yaws and syphilis are endemic. For example, a question was raised regarding the specificity of the RPR test in Vanuatu where surveys in 2000 found an unexpected syphilis prevalence of 2.4% in antenatal clinics women (RPR confirmed by TPHA). One of the authors (C. Capuano) was involved in the discussion, which concluded that in the absence of clinical symptoms, it was not possible to differentiate among the two infections (personal communications Dr. C. Capuano, 2001). Similarly, the inability to serologically differentiate yaws and syphilis can be an issue in countries where yaws was endemic in the past, and the prevalence of syphilis is known to be high. In 1992, Gershman et al. [41] also raised the issue following a large increase of serologically reported cases of syphilis in the Marshall Islands, a previously yaws endemic country where it was believed to have been eradicated in the late 1940s and early 1950s. New techniques such as the molecular methods reported in 2006 by Centurion-Lara et al. [42] to differentiate the three *T. pallidum* subspecies could be of great assistance in similar situations. However, such methods are expensive, complex, and they require more than the standard laboratory equipment found in the resources-limited countries still affected by yaws in the WPR. These constraints make such methods currently not affordable and of limited value for field use.

However, although prevention of yaws and syphilis is very different, both infections warrant treatment regardless of the ability to differentiate them. Thus, one can expect that in the WPR, diagnosis of yaws will continue to require the assessment of test results and clinical manifestations while carefully taking into account epidemiologic characteristics of yaws.

### 3.3. Yaws in Papua New Guinea

A nationwide mass treatment targeting the entire population of PNG took place between 1953 and 1958 under the Australian administration covering more than 90% of the population [20]. Many of the untreated individuals were residents of remote and isolated areas of the country where campaign reach was limited. The campaign was successful; only 2,352 cases were reported in 1959, and fewer than 500 cases were reported each year until 1973. A slight increase in the number of cases was recorded between 1973 and 1978. However, it did not exceed 1,000 cases per year [19], and most of them occurred in rural areas, in Bougainville, and around Rabaul in New Britain [18]. The disease was rare in the Highland districts and reported to be nonexistent in the Central Province and Port Moresby [40]. Consequently, yaws was removed from the national list of reportable diseases. The clinical appearance of yaws observed during this time period was milder (fewer lesions, plantar lesions, and bone involvement rarely observed) than in the 1950s, and these milder cases were described as attenuated cases [18, 40].

In 1964-65, a serological survey for treponemal disease involving 844 sera was conducted in the Eastern Highlands of PNG [43]. No clinical case of active yaws was found in the population, but some of the elderly were identified with clinical evidence of old yaws lesions. The seroprevalence reported by the authors varies from 3.9% to 79.2%. In 3 out of the 10 census units surveyed, the prevalence in children below 15 years ranged from 14.3% to 40%, and the authors concluded that the treponemal disease involved was yaws.

In December 1978, a mass campaign targeting the entire population was carried out on Karkar Island, Madang Province, after a rapid increase of yaws cases was recorded in the previous year [18]. Based on surveys carried out preceding the mass treatment, the estimated prevalence varied from 0% to 27%, and the prevalence of infectious cases (examined using dark-field microscopy) also varied from 2% to 22% on the island. Although the mass treatment reported to have covered above 90% of the island population, the island continued to report cases of yaws. In 1981, a more comprehensive survey reported 0.36% prevalence of infectious cases and 2% prevalence of noninfectious clinical cases in the surveyed villages. Cases with attenuated manifestation were also recorded during this outbreak, and a local doctor also noted that the response to PAM was poorer than in the 1950s. It is possible that penicillin was improperly stored and thus lost its potency.

In the early 1980s, clusters of cases were reported in the Provinces of East and West Sepik, East and West New Britain, New Ireland, and Milne Bay. The outbreak and following mass treatments in Yilui village, West Sepik Province, is well documented [19]. In Yilui village (population 509 based on 1980 census), the initial mass treatments in early 1984 found 193 clinical cases (more than 35% of the 1980 population). A follow-up treatment conducted six months later found 60 clinical cases, mainly children, including 49 clinical cases treated during the initial mass treatments. Serological tests were performed on a sample of clinical and nonsymptomatic individuals. A large proportion of nonsymptomatic individuals presented reactive VDRL tests (12 out of 19), suggesting that a large proportion of the village population had been exposed to the infection in the recent past, just prior to the mass treatments. Cases with attenuated symptoms were also reported in Yilui. In the Kiriwina and Trobriand Islands of Milne Bay Province, a small number of suspected cases among children aged 4 to 13 were reported in 1984 [21]. Approximately, 70% of the suspected clinical cases were serologically (VDRL) positive. No case presented the late stage of the disease.

In 1988, another outbreak was reported on Karkar Island in the village of Takia (population 1600) [22]. Among 632 children aged 0 to 15 years, 39 (6%) presented early lesions. All villagers were subsequently treated with benzathine penicillin. Blood samples were repeatedly collected from the clinical cases over 22 months following the initial treatment, and the authors found that 13% of the cases remained or became serologically positive again at 22 months despite the fact that many of the initial cases were treated multiple times. Clinical relapse among the initial clinical cases was reported to be 8% at 22 months. This incidence suggested that yaws cases in this area had decreased sensitivity to penicillin. However, the finding needs to be confirmed.

Between April 2000 and September 2001, the Nine Mile Clinic in Port Moresby identified 494 cases confirmed by serological tests (TPHA and VDRL) through clinic-based case detection [23]. The clinic serves approximately 20,000 individuals in the periurban population of northeast Port Moresby, where many live in squatter settlements with poor access to water and electricity. Yaws cases were seen at the clinic as early as 1995, and a yaws register was created in April 2000 after an increase in numbers was observed in 1999. The clinic also conducted a prevalence survey in 2001 at settlements near the clinic where prevalence was expected to be high. Among 227 children under the age of 17 examined, 33 had active yaws lesions as diagnosed by experienced medical staff. It is not clear whether these cases were later treated. Yaws cases had been rare in Port Moresby, and thus, this finding suggests that the cases observed at the clinic may have contracted yaws outside Port Moresby or from persons with yaws who migrated from other parts of the country. The population in Port Moresby is young and rapidly growing with a continuous migration of people throughout the country. With poor hygiene, crowded environments, and a large number of people previously unexposed to yaws, the population provides an ideal environment for yaws transmission [23].

Records on yaws in most areas of PNG are not available since the late 1980s. Therefore, it is difficult to assess the current distribution of the disease. However, as found in Port Moresby in 2000 and 2001, cases exist and transmission is still likely to be active. Our paper suggests that active transmission is currently occurring in various parts of PNG. However, it is important to note that no late-stage cases have been seen since the mass treatment in 1950s and many of the more recent cases presented with a small number of lesions.  Table 3 summarizes yaws reported cases and prevalence surveys in PNG from 1959 to 2001.

### 3.4. Yaws in Solomon Islands

A nationwide mass treatment campaign was carried out in the British Solomon Islands Protectorate between 1956 and 1958, covering all main islands and most of the other inhabited islands. An initial survey prior to the treatment campaign found the prevalence of active yaws cases above 14.5% [25]. The campaign was successful, and only a few cases were reported (from Malaita) following the campaign. The national yaws elimination project was then completed in 1963. A small number of cases were sporadically reported until 1970.

No cases were documented from 1970 until 1981 when an outbreak of cases was reported in the Western Province [16, 24, 44]. The cases were initially misdiagnosed as tropical ulcers until suspected cases presenting large leg ulcers were serologically confirmed as yaws in 1984 (serology done in Australia). Following the confirmation, a mass treatment of the entire population was carried out in the islands of Gizo, Vella la Vella, Ranonga, Simbo, New Georgia, Kolombangara, and North Choiseul. By the end of the campaign, 3,994 out of 29,235 persons examined (13.7%) were found to have active yaws [25]. The disease was more prevalent among children under the age of 15; 28% of children under 15 examined were diagnosed to have yaws [24]. Yaws was diagnosed clinically in the field by medical staff involved in the previous mass campaign in the 1950s. Follow-up visits in selected villages did not find any additional cases. A higher prevalence was reported in the islands of Vella la Vella, Ranonga, and Simbo. No case had been reported in the Shorthand Islands, despite its proximity to PNG [25].

In 1986, monthly reports from rural health clinics indicated a recurrence of yaws in areas of Western Province treated during the 1984 mass campaign. A sample survey was conducted in areas where a large number of cases were reported [16]. 83 definite cases (10%, papillomatous or ulceropapillomatous) and 68 suspected cases (8.2%, scanty macules and maculopapulomatous) were identified among 833 examined. Based on this finding, a treatment campaign was carried out in 1987 on the islands of Vella la Vella, Ranonga, Simbo, Kolombangara, New Georgia, and Gizo. Among the total of 24,216 persons treated, 2070 (8.5%) had clinical manifestations. Blood was collected from a random sample of the population, and 11% of 453 serum samples showed positive VDRL tests. In Simbo Island, where 47% of treated individuals (580 of 1,220) were clinically diagnosed to have yaws, 57% of cases presented primary lesions (a single raised lesion, often ulcerated), while 43% had secondary lesions (multiple ulcers and small papillomas). The majority of cases were under 15 years of age. Investigators also noted that cases in 1984 and 1987 presented milder attenuated symptoms (a few scanty lesions) than cases in 1950s who often presented with abundant large elevated papillomas. While tertiary yaws was common in the 1950s, it was not observed during the campaign in 1987.

No documents recording yaws between 1988 and 1998 seem to be publicly available. The Annual Health Report of 2007 compiled by the Ministry of Health provides some evidence of persistent foci existing in the country for the last decade [26].

Between 1998 and 2007, all 10 provinces reported cases of yaws, detected among patients attending primary health care facilities. No survey or mass treatment were reported to have been carried out since 1987. According to the report, the national incidence rate of yaws in 2007 estimated using the 2007 projected population of the Solomon Islands was reported to be 39 per 1000 person-years. Yaws cases accounted for 2% of all primary health care attendees in 2007. The incidence rate in 2007 was high in Makira (70.4 per 1000) and Guadalcanal (60.3 per 1000), and low in Isabel (19 per 1000) and Choiseul (17 per 1000). Based on the reported incidence rates and the 2007 projected provincial population [26], the absolute number of cases in 2007 was estimated to exceed 4,000 in Guadalcanal and Malaita. The disease incidence seems to have decreased nationally since 1998. However, the rates have fluctuated in the past, and the observed change in the rates may be due to changes in diagnosis and recording practices over the 10 year period. In 2007, a WHO consultant [45] noted that for the period from 1997 to 2006 the rate of clinical yaws reported was constantly highest in children aged between one and four years, and that in 2003, the rate increased with a marked rise in children and people aged five years and over. Since 2003, the rate has shown a downward trend, reaching its lowest point in 2006. There is no clear explanation for the increased rate observed in 2003.

It is also noted that cases of yaws are reported entirely on clinical grounds. In addition, data gathered since 1998 only include cases diagnosed in primary health care facilities. The figures may be overestimated if cases are misdiagnosed or overdiagnosed, or underestimated if many yaws cases do not seek care from the health system.

In 1984 and 1986, a woman in Australia who migrated from the Solomon Islands in 1975 was treated for tertiary stage yaws [46]. She visited the Solomon Islands in 1981 and suffered from likely secondary-stage yaws upon her visit. The authors speculated that she may have been reinfected during the visit leading to the destructive bony lesions consistent with a tertiary stage of yaws observed in 1984 and 1986. There has been no other report of imported yaws from the South Pacific in Australia or New Zealand.

As indicated by the 2007 national health report [26], it is likely that yaws is still found in most of the provinces in the Solomon Islands. However, more information on reported clinical cases, such as detailed descriptions of symptoms and serological status, is necessary to confirm whether the reported cases are true cases of yaws, and to identify the most affected areas.

Table 4 summarizes yaws reported cases and prevalence surveys in the Solomon Islands from 1956 to 2007.

### 3.5. Yaws in Vanuatu

In Vanuatu, the first mass campaign began in 1958. An initial survey treated approximately 94% of the resident population, and a followup was estimated to have covered 92% of the population [15]. About two-thirds of Tanna Island in the province of TAFEA was not treated during the initial treatment surveys due to refusal for political reasons [15]. The campaign was successful as confirmed by a survey in 1961. The estimated prevalence after the mass campaigns was reduced to 0.5 per 1000.

Throughout the 1970s, fewer than 100 cases were reported each year. In the late 1970s, an increase in cases in Tanna Island was reported. Further investigation of these cases revealed active yaws transmission in the island.

In 1981 and 1985, mass treatments were carried out in several villages in northwestern Tanna where clinical cases had been reported. Despite these efforts, yaws continued to reappear in Tanna, and a large-scale treatment campaign was planned in 1989. A survey conducted prior to the mass treatment found 116 (16.5%) clinically suspected cases out of 704 treatment participants in 13 villages and 1 school in Tanna [27]. Among the 97 clinically suspected cases, 34 showed VDRL titer above 1 : 4. Based on this finding, the mass treatment was planned to cover the entire population of Tanna, about 20,700 people. Approximately 90% of the population were treated, although children under the age of 3 months were excluded from examination and treatment. During the mass treatment, 348 clinically suspected cases were recorded. A large proportion of clinical cases (79.3%) were under the age of 15, and 32% of blood samples from 189 suspected cases had positive VDRL results. Following the campaign, only a few cases were reported in 1990. However, by 1992, a number of cases were reported from villages that previously had not participated in the treatment campaign. The laboratory reports between 1995 and 1998 also indicated that yaws was still not eliminated from the island [47].

In 2000, patients with suspicious lesions were reported on the island of Santo, SANMA province, where laboratory records showed no confirmed cases between 1995 and 1998. A preliminary investigation found 21 clinical cases in Central and South Santo including 20 cases serologically confirmed as yaws. From May to July 2001, a sample survey representative of the entire island was conducted. Among 273 individuals, 57 (20.9%) were serologically positive [47]. The majority (70%) of seropositives were males, and 40% were under 15 years of age. Following the survey, a mass treatment of the entire SANMA Province was carried out. People of all ages were eligible for treatment by benzathine penicillin. A total of 36,414 persons in SANMA Province were treated. The treatment coverage was estimated to be 92.4% based on the campaign registers and exceeded 100% based on the census population of 1999. During the treatment campaign, 251 clinically suspected cases were found and treated in 82 villages. A large proportion of clinical cases (78%) were under 15. Among suspected clinical cases who were later serologically tested, 40% (96 out of 230) had positive VDRL results. It was also noted that cases were concentrated in central and southern parts of the province.

The total estimated cost of the 2001 eradication campaign in SANMA Province reached USD 41,700 for a population of 36,414 or an estimated USD 1.15 per person [17]. 

In 2007, 789 cases were reported on a routine health report from the island of Tanna. A serological and clinical survey using a cluster sampling method was conducted in 2008 to assess the endemicity of yaws on this island. Among 306 individuals from whom blood samples were taken, 95 (31%) were positives for rapid plasma reagin (RPR) and/or rapid diagnostic test for syphilis (RDT). It is not stated whether the seropositive individuals showed clinical manifestations of yaws, had been treated for the disease in the recent past, or are active cases of yaws. Out of 473 individuals enrolled in the clinical survey, 55 (11%) had typical yaws lesions. All were below 15 years of age. Sixty six (14%) had skin lesions which may possibly have been yaws, and most of them (94%) were below 15 years of age. During this survey, only one tertiary case of yaws was seen in an adult, presenting with a mild saber tibia [28].

All cases reported since the 1970s were found in clusters on certain islands of TAFEA and SANMA Provinces. Follow-ups of treated cases could ensure prevention of further outbreaks. Active case detection and appropriate treatment may be necessary in some communities where a large number of cases were previously reported. Yaws has not been officially reported in the other provinces since the mass treatment campaigns in the late 1950s. However, the current situation has not been assessed and is unknown.

Table 5 summarizes yaws reported cases and prevalence surveys in Vanuatu from 1958 to 2008.

### 3.6. Yaws in Other Areas of the South Pacific

With the technical assistance of WHO and UNICEF, national yaws control programs were established in Fiji and Western Samoa (Samoa) in 1955, Gilbert and Ellice Islands (Kiribati and Tuvalu) in 1957, and Tonga in 1962. Initial surveys conducted by these national programs showed a varying degree of yaws endemicity in the region with the highest prevalence found in Fiji (28.81%). The results are summarized in Table 6 [29]. Based on the results of these surveys, selective or mass treatment with PAM was implemented, and the disease was nearly eliminated in these countries by the 1960s. Passive surveillance continued in these areas, and a very small number of cases were reported over the next few decades. Between 1969 and 1984, Fiji reported three cases, thought to be the very last in that country. The last reports of yaws in Tonga were 7 cases in 1976. In the Cook Islands, a nationwide eradication campaign in 1960 covering approximately 99% of the population found 17 cases of infectious yaws and 14 cases of noninfectious yaws [15]. No case has been documented in these areas of the South Pacific in recent years. Data on past yaws control efforts in remaining areas of the South Pacific are very limited.

### 3.7. Yaws in the Rest of the Western Pacific Region and in Neighboring Countries

#### 3.7.1. Western Pacific Region

The national control programs were established in Cambodia, Laos, Malaysia, and the Philippines in the 1950s. A significant reduction of prevalence to near elimination was found during the resurveys in the 1960s [29]. Little information on yaws after the 1960s is available from these countries. However, in 1985, Lo reviewed yaws control activities since the 1950s in Malaysia [48] and concluded that the program has been a success and that yaws disappearance from Malaysia could be expected. In the following years, only a small outbreak consisting of 10 active cases was reported in Baling, Malaysia in 1989 [49], and 6 imported cases of yaws were found in a family in Johor, Malaysia, in 1988 [50].

In Australia, the most recent information we found is from Garner et al. [51] who reported in 1972 results from a serological survey in the aboriginal population of the Northern Territory. They concluded that while no case of active treponemal infection was found, the prevalence of treponemal infection varied from 3.4% to 58% indicating that yaws, endemic syphilis, and probably venereal syphilis were present in the aboriginal population.

**Figure 1:**
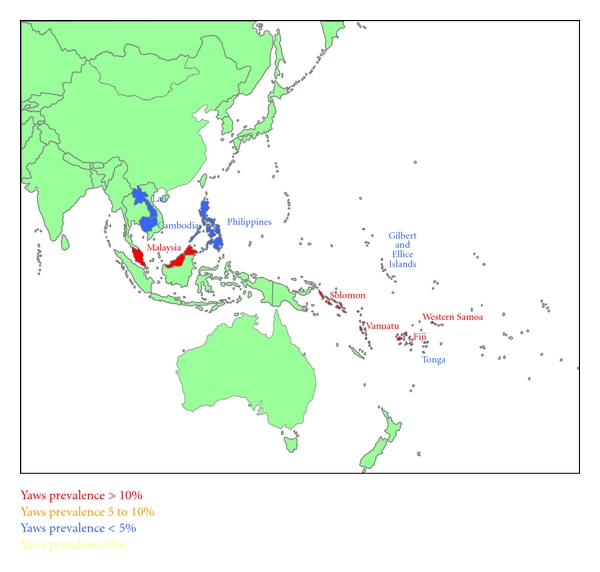
Yaws prevalence in the Western Pacific Region in the 1950s.

**Figure 2:**
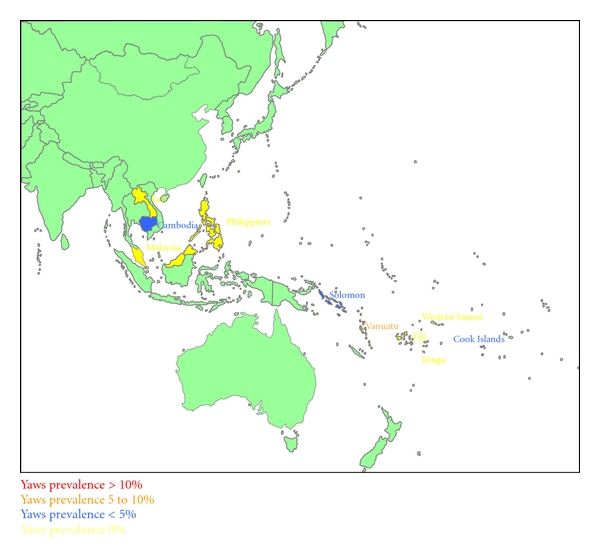
Yaws prevalence in the Western Pacific Region in the 1960s.

**Figure 3:**
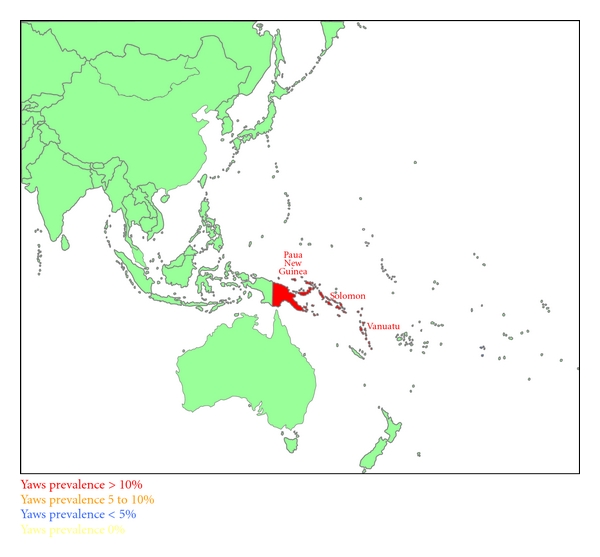
Yaws prevalence in the Western Pacific Region in the 1970s–1980s.

**Figure 4:**
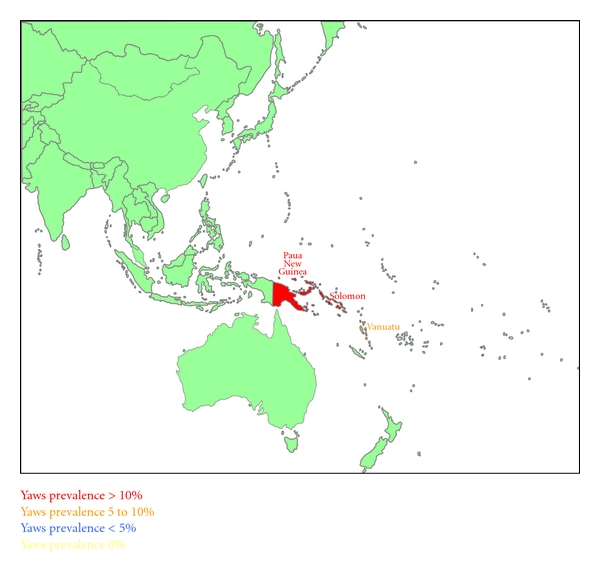
Yaws prevalence in the Western Pacific Region in the 1990s–present.

Figures 1, 2, 3, and 4 summarize the prevalence of yaws for the WPR from the 1950s to the present time.

#### 3.7.2. South East Asia Region

Yaws remains endemic in parts of Indonesia and Timor-Leste. In Indonesia, nearly 98% of cases reported in 2003 were from the provinces of East Nusa Tenggara, South East Sulewasi, Papua, and Maluku [52]. In Indonesia, yaws control became integrated with sexually transmitted disease control in 1980, further with the leprosy program in 2000, and then with the water and sanitation program in 2003. In the early 2000s, the reported annual incidence ranged from 2,000 to 4,500 cases [53]. In Timor-Leste, the disease has been reported from at least 6 of 13 districts in recent years [53]. Yaws control is currently part of an infectious disease control program targeting leprosy, lymphatic filariasis, and soil-transmitted helminthiasis. The estimated incidence of yaws in Timor-Leste ranges from 500 to 1,000 cases per year.

In both Indonesia and Timor-Leste, the assessment of incidence relies on reports from primary health facilities; therefore, the reported incidences may not be accurate due to underreporting or overdiagnosis. Obtaining greater political commitment and sufficient resources has been a key challenge for strengthening the existing yaws control programs in these countries.

India declared yaws elimination in 2006, after an absence of yaws cases since 2004 [30]. The Yaws Eradication Programme in India commenced in 1996 as a pilot program following the development of an eradication strategy by the National Institute of Communicable Disease and was expanded to all endemic areas of the country in 1999. The strategy consisted of two core activities: (1) active case detection and treatment of cases and contacts with long-acting penicillin and (2) community mobilization to raise awareness through Information Education Communication programs. The program was phased into three stages (attack, maintenance, and elimination/eradication) and had well-defined criteria for elimination (nil reporting of new infectious cases) and eradication (noseroactivity to RPR or VDRL in children below 5 years of age after achieving “nil” reporting for three years) as well as certification processes [53]. Four appraisals were conducted in 2000, 2002, 2004, and 2006, each conducted by an independent team consisting of a public health expert, a dermatologist, and a microbiologist. In addition to careful and detailed planning of program activities, factors thought to have contributed to the success include high-level political commitment maintained throughout the campaign, advocacy for sustained commitment, and the availability of resources. Rigorous active case detection, where all suspected cases and contacts were treated, was conducted biannually. Supportive monitoring and supervision of peripheral health workers also assured the high quality of case detection and helped to maintain their motivation throughout the campaign. Following the declaration of elimination in India, the WHO South-East Asia Region has set 2012 as a target of yaws elimination from Indonesia and Timor-Leste.

## 4. Conclusions

After the successful eradication campaigns of the 1960s, the primary health care systems were supposed to give the last push towards eradication of yaws. However, a combination of various factors including poor political commitment and limited funding resulted in a missed opportunity and disappointed the hopes raised half a century ago.

Yaws presents new challenges such as poor awareness and knowledge among health care workers, unknown epidemiological situation, and attenuated clinical forms of the disease. These challenges, combined with the current competing priorities in the public health arena, require new, innovative, and country-tailored approaches.

Recent experience in India as well as past experiences in the South Pacific clearly demonstrate that with a well-designed elimination strategy based on reliable epidemiological data and sustained high-level political commitment, it is possible to eliminate yaws.

The increased attention required to address the resurgence of yaws in the affected countries does not necessarily mean increased resources. In view of the many health priorities and the human and financial resources constraints, it rather calls for new and innovative approaches. Today, utilization of existing programs with similar primary target population (i.e., children under 15 years of age) in implementing yaws control activities may provide a cost-effective option. For example, yaws screening and detection could be included in school-based health promotion programs such as a deworming program for soil-transmitted helminthiasis or dental surveys. Cost-effective strategies in identifying cases, in providing treatment, and in tracing all contacts need to be identified at country level in order to take into account logistical constraints, resources available locally, and other health priorities in the affected countries. Many opportunities to revitalize yaws control activities already exist at country level: Maternal and Child Health clinics and their related activities, water and sanitation programmes, or school-based programmes are such examples. One way to approach yaws control might be to address it in the broader context of the Primary Health Care (PHC) where lessons learnt from past failures would be used to avoid repeating the same mistakes. Yaws control activities could be powerful tools for revitalizing and/or strengthening PHC in endemic countries, especially if they use the momentum usually created by mass campaigns, which are still necessary to bring down the level of transmission in highly endemic areas. Such an approach may mean that yaws eradication will take longer than with repeated mass campaigns, but considering the competing health priorities and other challenges faced by the affected countries, it might be a more sustainable and realistic strategy. It also means that before a mass campaign is considered due consideration must be given to the role of the PHC system before, during, and after the campaign is carried out.

However, at this stage it is of tremendous importance to get a better understanding of the current epidemiological situation through a strong and systematic assessment of yaws in the Pacific in general and in PNG, Vanuatu, and Solomon in particular. Only then, sound strategies towards elimination of yaws can be developed, and the work started more than half a century ago can be completed.

## Figures and Tables

**Table 1:** WHO recommended treatment strategy by disease prevalence [2].

Approximate prevalence of clinically active yaws in the community	Endemicity classification	Recommended treatment
High (>10%)	Hyperendemic	Entire population of the community (total mass treatment)
Medium (5–10%)	Mesoendemic	All active cases, all children under 15, and obvious contacts (juvenile mass treatment)
Low (<5%)	Hypoendemic	All active cases and all household and other obvious contacts (selective mass treatment)

**Table 2:** Estimating seroreactor prevalence from population prevalence [2].

Percentage of population with active yaws	Percentage of seroreactors
1-2	8.5
11–15	54.0
16–20	71.0
21–30	77.5

**Table 3:** Summary of yaws reported cases and prevalence surveys in PNG since 1959.

Area	Year	Prevalence	N*	Activity type	Reference
All areas	1959-60	2352 cases	—	Reports	Garner et al., [19]
Eastern highlands	1964-65	29.3%	844	Serological survey	Garner and Hornabrook, [43]
All areas	~1973	Fewer than 500 cases annually	—	Reports	Garner et al., [19]
All areas	1973–78	500*∼*1000 cases annually	—	Reports	Garner et al., [19] Reid, [18]
Karkar Island, Madang Province	1978	Clinical 0–27% Infectious 2–20%	~1800	Preliminary survey prior to mass treatment	Reid, [18]
Karkar Island	1978	Clinical 4.7%	—	Mass treatment of 92–95% of Karkar Island population	Reid, [18]
Karkar Island	1981	Infectious 0.36% Noninfectious 2%	—	Survey conducted in randomly selected villages	Reid, [18]
Yilui village, West Sepil Province	1984	204 clinical cases	—	Screening concurrent with mass treatment of all villagers	Garner et al., [19]
Kiriwina Island, Trobriand Island	1984-85	34 suspected cases 33 out of 49 blood specimen were VDRL positive	—	Cases reported from health centers	Duncan and Alto, [21]
Marup village, Karkar Island, and Madang Province	1988	Early lesions 6%	632	Survey of children (0–15 yo) following outbreak, followed by mass treatment of all villagers Report of decreased sensitivity to penicillin	Backhouse et al., [22]
Periurban settlements of Port Moresby	2000-01	494 clinical cases	—	Cases detected at the 9 Mile Clinic	Manning and Ogle, [23]
Periurban settlements of Port Moresby	2001	Clinical cases 14.5%	227	Children under 17 at four settlements (convenient samples) near the 9 Mile Clinic	Manning and Ogle, [23]

*The number of persons examined/screened.

**Table 4:** Summary of yaws reported cases and prevalence surveys in Solomon Islands since 1956.

Area	Year	Prevalence	N*	Activity type	Reference
All main islands and most of inhabited islands	1956–58	Active 14.5% Infectious 2.9%	112, 700 (estimated)	Screening concurrent with whole population mass treatment	Alemaena, [25]
Western Solomon Islands	Late 1960s–1970	Unconfirmed cases reported	—	—	Alemaena, [25]
Western Province	1984	Active 13.7%	29,235	Screening concurrent with mass treatment of Western Province Cases reported in Vella la Vella, Ranonnga, and Simbo	Alemaena, [25]
Australia	1984	—	—	Case study of immigrant woman with tertiary yaws from Solomon Islands	Wallace and Ellis, [46]
Western Province	1986	Definite yaws^1^ 10% Suspected yaws^2^ 8.2%	833	Sample survey, representative of Vella la Vella, Ranonga, and Simbo	Fegan et al., [16]
Western Province	1987	Active 8.5% 11% of 453 cases was VDRL positive	24,216	Screening concurrent with mass treatment of Western Province (except Choiseul)	Fegan et al., [16]
All areas	1998	59.1 per 1,000^3^		Clinic/hospital reports	MOH, Solomon Islands, [26]
All areas	1999	49.5 per 1,000		Clinic/hospital reports	MOH, Solomon Islands, [26]
All areas	2000	57.4 per 1,000		Clinic/hospital reports	MOH, Solomon Islands, [26]
All areas	2001	46.8 per 1,000		Clinic/hospital reports	MOH, Solomon Islands, [26]
All areas	2002	49.0 per 1,000		Clinic/hospital reports	MOH, Solomon Islands, [26]
All areas	2003	65.4 per 1,000		Clinic/hospital reports	MOH, Solomon Islands, [26]
All areas	2004	50.7 per 1,000		Clinic/hospital reports	MOH, Solomon Islands, [26]
All areas	2005	47.6 per 1,000		Clinic/hospital reports	MOH, Solomon Islands, [26]
All areas	2006	42.2 per 1,000		Clinic/hospital reports	MOH, Solomon Islands, [26]
All areas	2007	39.2 per 1,000		Clinic/hospital reports	MOH, Solomon Islands, [26]

*The number of persons examined/screened.

^1^Definite cases: papillomatous or ulceropapillomatous.

^2^Suspected cases: scanty macules and maculopapulomatous.

^3^Rates of disease are calculated per 1,000 populations by dividing the number of cases of disease reported by the population in each age group and multiplying by 1,000.

**Table 5:** Summary of yaws reported cases and prevalence surveys in Vanuatu since 1958.

Area	Year	Prevalence	N*	Activity type	Reference
All areas except Tanna Island	1958	—	94% of population (estimated)	Mass treatment	Geizer, [15]
Tanna Island, TAFEA	1985	495 cases reported	—	Cases reported from clinic/hospital, followed by mass treatment of villages in North Western Tanna	Harris et al., [27]
Tanna	1988-89	Clinical 16.5% 34 of 97 clinical cases were VDRL positive	704	Preliminary investigation prior to mass treatment 13 villages and 1 school	Harris et al., [27]
Tanna	1989	Clinical 1.9% 32 of 189 clinical cases were VDRL positive	18,213	Screening concurrent with mass treatment of ~90% of Tanna Island population	Harris et al., [27]
Santo, SANMA	2000	21 clinical cases 20 out of the 21 cases VDRL positive	—	Preliminary investigation prior to population sample survey	Yaws mass campaign SANMA province, final report, [47]
Santo, SANMA	2000	VDRL positive 20.9%	273	Sample survey, representative of Santo Island	Yaws mass campaign SANMA province, final report, [47]
SANMA	2001	Clinical 0.7% (96 out of 230 clinical cases were VDRL positive)	36,414	Screening concurrent with mass treatment of 92.4 + % of SANMA province population	De Noray et al., [17]
Tanna	2007-08	789 suspected cases in 2007 187 suspected cases in 2008	—	Health center/hospital-based cases	Yaws prevalence survey, Tanna Island, Report, [54]
Tanna	2008	31% positive RDT and/or RDT	306	Sample survey (30 by 7)	Fegan et al., [28]

*The number of persons examined/screened.

**Table 6:** Prevalence of yaws pre- and postmass treatment in the 1950s and 1960s in Pacific countries [26].

Country/area	Prevalence of active yaws (infectious yaws) %	
	Initial survey in the 50s	Resurvey in the 60s
Cook Islands	Not available	0.21 (0.01)
Fiji	28.81 (5.86)	0.00
Gilbert and Ellice Islands	2.21 (0.56)	—*
Tonga	2.20 (0.03)	0.01 (0.01)
Western Samoa	11.0 (2.90)	0.00 (0.00)

*No survey carried out or considered necessary.
